# Perceived Immune Fitness, Individual Strength and Hangover Severity

**DOI:** 10.3390/ijerph17114039

**Published:** 2020-06-05

**Authors:** Aurora J. A. E. van de Loo, Nikki Kerssemakers, Andrew Scholey, Johan Garssen, Aletta D. Kraneveld, Joris C. Verster

**Affiliations:** 1 Institute for Risk Assessment Sciences (IRAS), Utrecht University, 3584CM Utrecht, The Netherlands; a.j.a.e.vandeloo@uu.nl; 2 Division of Pharmacology, Utrecht Institute for Pharmaceutical Sciences (UIPS), Utrecht University, 3584CG Utrecht, The Netherlands; n.kerssemakers@students.uu.nl (N.K.); j.garssen@uu.nl (J.G.); a.d.kraneveld@uu.nl (A.D.K.); 3 Centre for Human Psychopharmacology, Swinburne University, Melbourne, VIC 3122, Australia; andrew@scholeylab.com; 4 Nutricia Research, 3584CT Utrecht, The Netherlands

**Keywords:** alcohol, hangover, immune fitness, fatigue, predictors

## Abstract

Various factors may contribute to alcohol hangover severity. The purpose of the current investigation was to evaluate the possible impact of alcohol consumption patterns, perceived immune status, and baseline fatigue on hangover severity. A survey was completed by a convenience sample of *N* = 199 Dutch students who reported on their latest past month’s heavy drinking occasion, including subjective intoxication (perceived drunkenness) and next-day hangover severity, which were rated on single-item scales ranging from 0 (absent) to 10 (extreme). In addition, perceived (momentary) immune fitness was assessed, and the Checklist Individual Strength (CIS) was completed to assess baseline fatigue. The analysis revealed that instead of the amount of alcohol consumed or estimated blood alcohol concentration, it appeared that subjective intoxication (i.e., level of drunkenness) was the most important determinant of alcohol hangover severity. Especially in men, albeit modest, it was perceived that immune fitness also significantly contributed to the level of hangover severity experienced.

## 1. Introduction

The alcohol hangover is the most commonly reported negative consequence of increased alcohol consumption [1] and has been defined as ‘the combination of negative mental and physical symptoms which may be experienced the day after a single episode of alcohol consumption, starting when blood alcohol concentration (BAC) approaches zero’ [2,3]. The hangover is often accompanied by impairments of cognitive and psychomotor performance [4,5,6] and mood changes [7], which may negatively impact daily activities such as driving [8,9] or work performance [10].

Previous research focused on various factors that may predict hangover severity. For example, both state and trait mood appeared to have a limited impact on hangover severity [11]. Other studies found that consuming alcoholic beverages with high levels of congeners (i.e., substances other than ethanol, produced during fermentation)—may aggravate hangover severity [12]. More commonly, research investigated the possible impact of alcohol consumption variables (i.e., quantity, frequency measures) in relation to hangover severity. Such studies revealed that the amount of alcohol consumed or estimated BAC was not the most important predictors of hangover severity. Rather, subjective intoxication, i.e., the level of reported drunkenness, was showns to be a more important predictor of hangover severity [11,12,13,14].

Several review papers have suggested various physiological and subjective factors that contribute to the pathology of alcohol hangovers [15,16,17,18]. Over the past 10 years, there has been a growing consensus that the alcohol hangover may be provoked by an inflammatory response elicited by alcohol intake [19,20].

Immune fitness can be defined as the capacity of the body to respond to health challenges (such as infections and/or fever) by activating an appropriate immune response in order to promote health and prevent and resolve disease, which is essential for improving quality of life. Perceived immune fitness is the individual subjective judgment of this capability. It has been shown that the past year’s immune status is related to the susceptibility to hangovers [21]. Van de Loo et al. reported that across all estimated BAC levels, subjects who reported having hangovers regularly had a significantly poorer past year’s immune status compared to drinkers that claimed to be hangover resistant. However, in a subsequent study, van de Loo et al. [22] observed that momentary (real-time) perceived immune fitness did not correlate significantly with overall hangover severity, after controlling for estimated BAC. Another recent study also revealed that mental and physical fitness were not significantly contributing to the regression analysis that aimed to predict hangover severity [14,23]. Instead, subjective intoxication was again the strongest determinant factor of hangover severity.

Physical fitness and exercise may strengthen immune fitness [24]. However, the number of studies investigating whether baseline levels of mental resilience or physical activity levels are predictive of hangover severity is limited. Physical activity levels have been shown to be reduced during the hangover state [25]. However, the limited number of studies that have examined physical performance revealed no significant differences between assessments made on hangover days and alcohol-free control days [24,25,26,27,28].

The current survey was conducted among a sample of Dutch students to further investigate the relationship between hangover severity and perceived immune fitness (state), alcohol consumption, and physical strength.

## 2. Materials and Methods

In September 2019, a survey was conducted among a convenience sample of *N* = 199 Dutch students (*n* = 54 men and *n* = 145 women). They were recruited at Utrecht Science Park by the investigator. Subjects were included if they were between 18 and 25 years old. There were no exclusion criteria, but subjects had to consume alcohol to participate in the survey. When inviting subjects to participate, they were first asked whether or not they consume alcohol. If they answered yes, they were asked to complete the survey. Although a minority, no formal recordings were kept of subjects who did not consume alcohol or who refused to participate. Subjects completed the paper-pencil survey in the presence of the investigator (N.K.), at the place of recruitment (university canteens or student study facilities). Participation was anonymous, and they received no financial reward for participation. The Ethics Committee of the Faculty of Social and Behavioral Sciences of Utrecht University granted ethical approval (approval code FETC17-061), and informed consent was obtained.

As part of the survey, demographics were recorded, including age, sex, height, and weight. Body mass index (BMI, kg/m^2^) was calculated using this information. Alcohol consumption was recorded using questions taken from the Quick Drinking Screen [29,30], including information on their usual alcohol intake on a drinking occasion and consumption variables concerning their past month’s heaviest drinking occasion. These included questions about the amount of alcohol consumed and the drinking time. The survey contained guidance about standard drinking sizes and how to convert, for example, bottles of wine into standardized alcohol units, containing 10 g of ethanol each in The Netherlands. Together, with information on sex and body weight and using an updated Widmark equation [31], this allowed to calculate estimated BAC. Subjective intoxication was rated on a single item scale, ranging from 0 (sober) to 10 (very drunk) [32,33]. Hangover severity was rated on a single item scale, ranging from 0 (absent) to 10 (extreme) [34]. A single item scale that provides an overall rating for hangover severity was chosen as it better reflects the overall hangover experience than scores of scales that aggregate a limited number of hangover symptoms [34].

Current perceived immune fitness was assessed using a 1-item scale ranging from 0 (very poor) to 10 (excellent) [35,36]. The Dutch version of the Checklist Individual Strength (CIS) [37,38] was completed to evaluate past month’s subjective fatigue and related constructs. The scale consists of 20-item that can be scored on 7-point Likert scales with the anchors ‘yes, true’ (1) and ‘no, not true’ (7). A sum score can be computed (with some items having reversed scoring) and four subscales, assessing ‘fatigue,’ ‘concentration,’ ‘motivation,’ and ‘physical activity.’ Higher scores suggest more subjective fatigue and more concentration problems, and less motivation and less physical activity. A fatigue score > 27 suggests abnormal fatigue and a score > 37 indicates severe fatigue [39].

Statistical analyses were conducted with SPSS (IBM Corp. Released 2013. IBM SPSS Statistics for Windows, Version 25.0. IBM Corp: Armonk, NY, USA). Mean and standard deviation (SD) were computed for each variable. Depending on whether the data had a normal distribution, paired t-tests or Independent Samples Mann–Whitney U tests were used to compare men and women.

Partial correlations, corrected for estimated BAC, were computed to evaluate associations between study outcomes and hangover severity. The results of the analyses were considered statistically significant if *p* < 0.05. A linear stepwise regression analysis was conducted to determine which of the variables assessed in this survey was a significant predictor of hangover severity. The regression analysis was conducted for the sample as a whole, and for men and women separately.

## 3. Results

N = 199 participants completed the survey. Their demographics, drinking variables, and other study outcomes are summarized in Table 1 and Table 2.

Significant sex differences were observed in drinking behavior. As expected, men consumed more alcohol than women and reached a higher estimated BAC. From the average estimated BAC (0.22%), it is evident that this was a relatively heavy drinking sample. No sex differences were found on the CIS assessments of individual strength.

**Figure 1:**
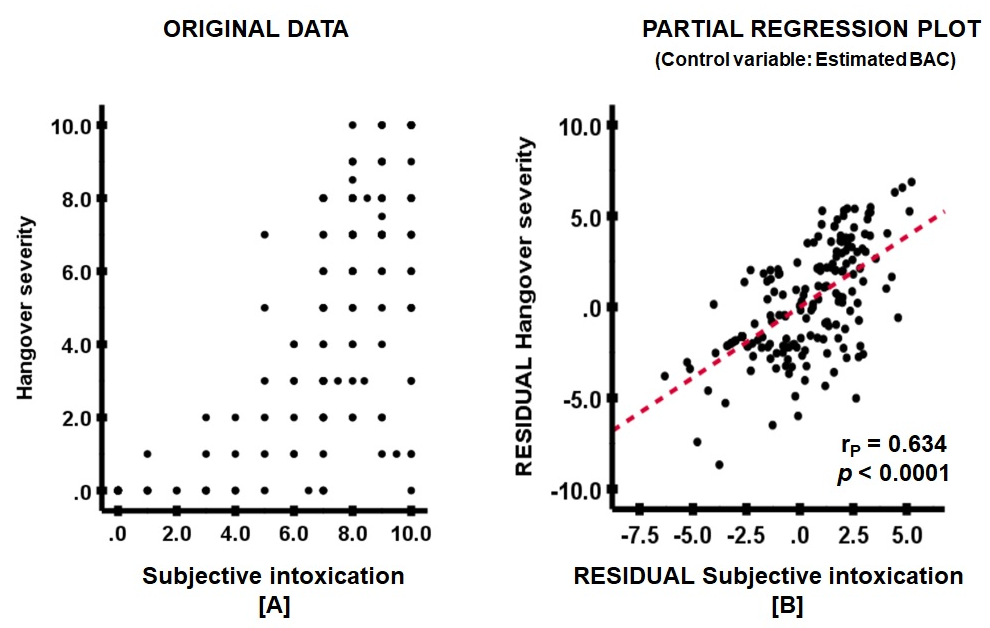
The association between subjective intoxication and overall hangover severity. A partial correlation (r_p_) was computed between hangover severity and ethanol elimination rate. (**A**) shows the original data, (**B**) the partial regression plot. The red line represents the partial correlation. Abbreviation: BAC = blood alcohol concentration.

Partial correlations, correcting for estimated BAC, revealed no significant associations between hangover severity and perceived immune fitness, nor with any outcome of the CIS. In contrast, a strong positive partial correlation was observed between hangover severity and subjective intoxication (Figure 1).

A stepwise regression analysis revealed that the amount of alcohol consumed and estimated BAC were no relevant predictors of overall hangover severity. Instead, a model that explained 54.3% of variance was obtained, with subjective intoxication as the most important contributing factor to hangover severity (50.9%), followed by significant but less strong contributions of the variables, age (1.6%), perceived immune fitness (1.0%), and fatigue (0.8%).

When conducting the regression analysis for men only, a model was obtained explaining 45.1% of variance in overall hangover severity, with subjective intoxication as the most important contributing variable (40.0%), followed by perceived immune fitness (5.1%). When conducting the regression analysis for women only, a model was obtained explaining 51.5% of variance in overall hangover severity, with subjective intoxication as the single contributing variable.

## 4. Discussion

In line with previous research, the amount of alcohol consumed and estimated BAC did not contribute to the models predicting hangover severity. However, the analyses confirmed previous studies that showed that subjective intoxication is a very strong predictor of hangover severity [11,12,13,14,40,41]. At a group level, there is a high correlation between the amount of alcohol consumed and subjective intoxication [32]. However, individual drinkers vary considerably in the level of subjective intoxication when consuming the same amount of alcohol. This may explain why the amount of alcohol consumed predicts subjective intoxication, but not hangover severity. The effect of alcohol consumption, i.e., the levels of subjective intoxication, is significantly related to hangover severity.

Perceived immune fitness had a modest contribution to overall hangover severity, which was most pronounced in men (5.1%). In contrast, women’s perceived immune fitness did not significantly contribute to the model explaining hangover severity. The relationship between susceptibility to hangovers and immune fitness was reported previously [20]. However, in contrast to previous findings [21], the current data also suggest a significant relationship between perceived immune fitness and hangover severity. We have no clear explanation for the observed sex difference. Sex differences in hangover symptom severity, albeit modest after correcting for drinking volume, have been reported previously [42]. However, immune function and susceptibility to immune-related diseases differ between men and women [43,44], which may have been reflected in a differential perception of the immune fitness of men and women in the current study. More research into sex differences in perceived immune fitness is warranted to further elucidate possible sex differences.

In contrast to our hypothesis, baseline fatigue levels did not relevantly contribute to hangover severity (0.8%). Interestingly, the sample scored relatively high on the CIS fatigue scale. The group average of 29.6 was higher than the cut-off score 27, indicating abnormal fatigue in the general population [39]. The lifestyle of students, including irregular sleep and sleep deprivation, may account for this. Indeed, research revealed that about 70% of students attain insufficient sleep [45]. Previous research has shown that sleep duration and quality after consuming alcohol could significantly impact next-day hangover severity [26,46,47,48,49]. Sleep was, however, not assessed in the current study. Future studies should also investigate the specific relationship between subjective intoxication and subsequent sleep duration and quality, and infer whether fatigue while drinking has a relevant impact on next-day hangover severity.

Strengths of the study include that previous observation was confirmed in a high-level drinking sample, and that potential sex differences were evaluated. However, the survey collected data retrospectively, and as such, recall bias may have affected the data. Another limitation was the fact that only a relatively small convenience sample consisting of young students was investigated. Further research in larger samples should investigate how these observations translate to other age groups with differential alcohol consumption levels. Moreover, the state immune status was not assessed. Future studies should investigate this, for example, by implementing the Immune Status Questionnaire [35] in addition to the momentary assessments of immune fitness. Finally, the current observations rely entirely on self-report. In future studies, the assessments of perceived immune fitness should be compared to the presence of objective biomarkers of immune functioning (e.g., cytokines) to evaluate to what extent perceived immune fitness adequately reflects objectively assessed immune functioning.

## 5. Conclusions

This study confirms that instead of the amount of alcohol consumed, subjective intoxication is the most important determinant of alcohol hangover severity. The data suggest that immune fitness, albeit modest, also contributes to the experienced level of hangover severity. Future studies in larger samples should further investigate the role of the immune system in the pathology of the alcohol hangover.

## Figures and Tables

**Table 1:** Demographics and study outcomes.

	Overall	Men	Women	*p*-Value
Perceived immune fitness	7.2 (1.7)	7.7 (1.7)	7.0 (1.6)	0.005 *
Demographics				
*N*	199	54	145	
Age	19.6 (1.7)	20.7 (1.9)	19.2 (1.5)	0.000 *
BMI (kg/m^2^)	21.9 (3.4)	23.0 (5.0)	21.5 (2.4)	0.021 *
Alcohol per week (units)	10.6 (14.4)	18.5 (19.7)	7.7 (10.6)	0.000 *
Checklist Individual Strength				
Overall	69.2 (16.7)	67.9 (16.2)	69.2 (16.7)	0.561
Fatigue	29.6 (8.6)	27.8 (8.7)	30.2 (8.5)	0.105
Concentration	18.3 (5.7)	18.4 (5.6)	18.3 (5.7)	0.821
Motivation	11.4 (3.8)	11.4 (3.9)	11.4 (3.8)	0.955
Physical activity	9.6 (3.5)	10.0 (3.3)	9.5 (3.5)	0.217

Mean and SD (between brackets) are shown. Significant differences between men and women (*p* < 0.05) are indicated by *. Abbreviations: BMI = body mass index.

**Table 2:** Past month heaviest drinking occasion.

	Overall	Men	Women	*p*-Value
Number of alcoholic drinks (units)	12.3 (9.4)	18.5 (10.6)	10.1 (7.9)	0.000 *
Drinking time (h)	5.2 (3.5)	6.5 (3.5)	4.7 (3.3)	0.001 *
Estimated BAC (%)	0.22 (0.18)	0.25 (0.17)	0.20 (0.18)	0.023 *
Subjective intoxication	6.0 (3.6)	7.4 (3.2)	5.5 (3.6)	0.000 *
Overall hangover severity	4.0 (3.5)	5.5 (3.5)	3.5 (3.4)	0.000 *

Mean and SD (between brackets) are shown. Significant differences between men and women (*p* < 0.05) are indicated by *. Abbreviations: BAC = blood alcohol concentration.
